# Total Hip Arthroplasty or Arthroscopy for Pigmented Villonodular Synovitis of the Hip: A Retrospective Study with 3‐Year Follow‐Up at Minimum

**DOI:** 10.1111/os.13707

**Published:** 2023-04-24

**Authors:** Tao Li, Lu Mei, Yang Xu, YuanYiNuo Cao, XiaoJun Shi, Gang Chen, Jian Li

**Affiliations:** ^1^ Department of Orthopedics, Orthopedic Research Institute, West China Hospital Sichuan University Sichuan P. R. China; ^2^ West China School of Nursing, Sichuan University/Department of Orthopedics, West China Hospital Sichuan University Sichuan P. R. China

**Keywords:** Arthroscopy, Pigmented villonodular synovitis, Total hip arthroplasty

## Abstract

**Objective:**

Pigment Villonodular synovitis of the hip, a rare pain proliferation of the synovium, was treated successfully with total hip arthroplasty and arthroscopy. Most recent results come from small case series with no study comparing arthroscopy and arthroplasty. In this study, we aimed to show and compare the clinical outcomes of arthroscopy and total hip arthroplasty (THA) in pigment Villonodular synovitis of the hip.

**Methods:**

This was a retrospective clinical trial with data from patients with pigment Villonodular synovitis of the hip between 2010 and 2019. The study included 17 patients in the THA group, and 20 patients in the arthroscopy group. The clinical outcomes were evaluated at 3, 6, and 12 months, at 1 and 2 years, and every 5 years afterward. The clinical efficacy was measured using the Harris hip scores (HHSs) and visual analogue scale (VAS) score.

**Results:**

The mean HHS improved from 45.24 ± 10.36 to 78.94 ± 19.11 in the THA group (*t* = −6.394, *P* = 0.000) and 45.30 ± 11.08 to 71.60 ± 19.78 (*t* = −5.187, *P* = 0.000) in the arthroscopy group from pre‐operation to the final follow‐up. There is no significant difference between the two groups (*t* = 1.051, *P* = 0.301). The mean VAS improved from 3.65 ± 0.79 to 0.35 ± 0.70 (*t* = 12.890, *P* = 0.000) in the THA group and 4.05 ± 0.94 to 1.35 ± 1.79 (*t* = 5.979, *P* = 0.001) in the arthroscopy group postoperatively. There is no significant difference between the two groups (*t* = 1.329, *P* = 0.193). Recurrence of PVNS was diagnosed in four patients (20%) of the arthroscopy group and they underwent THA after arthroscopy, and the mean interval was 44.25 ± 6.98 months. All patients reached level 5 muscle strength by the final follow‐up. All the patients' buckling ranges were over 105 degrees. Their internal and external hip rotation was over 15 degrees. Their hip adduction was over 20 degrees, and abduction over 30 degrees.

**Conclusion:**

Both THA and arthroscopy in the setting of PVNS can improve patients' function and lead to a low rate of local recurrence. By selecting patients well for each approach, one can expect a reasonable result.

## Graphical abstract

THA and arthroscopy in the setting of PVNS can improve patients' function and lead to a low rate of local recurrence. In well‐selected patients for each approach, one can expect a reasonable result.

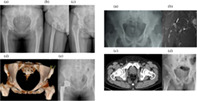

## Introduction

Pigmented Villonodular synovitis (PVNS), which was first described by Chassaignac in 1852 and characterized by Jaffe in 1941, is characterized by proliferation of the synovial tissue and severe joint destruction.^1^, ^2^ It typically presents with monoarticular pain, swelling, coffee‐colored joint fluid on needle aspiration, and stiffness.^3^, ^4^, ^5^, ^6^ The knee is the most commonly involved, but prior research suggests that the hip joint is involved in 15% of cases.^2^, ^6^ PVNS often leads to lytic lesions and cartilage destruction on both sides of the hip joint, especially in the area close to the synovial membrane, unlike the knee joint, where the shared space is often preserved.^1^, ^7^ Initial radiographs are customary in the early stage of PVNS. For the hip joint, disease diagnosis is often delayed due to the difficulty of palpation on physical examination and the difficulty of detecting imaging changes on early X‐rays, so many patients are symptomatic for years before diagnosis (with an average of 4–5 years for hip PVNS).^3^, ^6^, ^8^

The standard treatment of PVNS involves surgical resection combined with possible radiotherapy.^6^ However, PVNS has a high recurrence rate and is a locally destructive process even after surgery.^4^ At present, it is agreed that surgical treatment should be adopted, but there is no unified standard for the selection of surgical methods, and the therapeutic effect of different surgical methods is also controversial. Arthroscopy or arthroplasty combined with synovectomy has been the treatment of choice for patients with PVNS of the hip. It has been reported that total hip arthroplasty (THA) treatment lowers disease recurrence rates and has good therapeutic effects for patients who have serious damage to the hip, such as osteoarthritis patients with subchondral bone injury. However, available reports show poor results after THA in these young patients, and they may require more than one THA due to the expiration date of the hip joint prosthesis. Synovectomy under arthroscopy has become another choice. Arthroscopy can remove the affected synovial membrane to prevent further loss of hip function, but full removal of the lesion may not be possible and may lead to recurrence. The selection of appropriate surgical methods presently needs to be considered according to patients' conditions. The observation of long‐term clinical follow‐up results of different surgical methods can be used as a reference.^1^, ^2^, ^6^, ^8^, ^9^ However, there is a paucity of data describing the mid‐ to long‐term outcomes comparing arthroscopic synovectomy and THA. Therefore, the aims of the study were as follows: (i) to investigate the functional outcomes of PVNS patients undergoing surgical treatment (arthroscopy or arthroplasty) and to analyze mid‐ to long‐term outcomes after surgical treatment, including recurrence and complications; and (ii) to compare which surgical methods could lead to better clinical outcomes.

## Methods

This research study was conducted retrospectively in the local institution from data obtained for clinical purposes between 2010 and 2019. This research was approved by the local institutional review board (2019610).

The inclusion criteria were as follows: (i) inpatient patients with PVNS confirmed by pathological examination; (ii) patients undergoing THA or arthroscopy for histologically confirmed PVNS; and (iii) minimum 3‐year follow‐up.

The exclusion criteria were as follows: (i) patients with various metabolic bone diseases, such as incomplete osteogenesis and osteomalacia.

Finally, 19 patients undergoing THA and 22 patients undergoing arthroscopy were identified. Four patients were excluded due to follow‐up length (less than 24 months, two patients in the THA group and two in the arthroscopy group). Four patients in the arthroscopy group reached the criteria for THA and underwent THA.

### Indication of Operation

In general, we used arthroscopic surgery if the specific diagnosis of PVNS was in question since we used arthroscopic treatment to obtain biopsy specimens under these circumstances: (i) degree of joint destruction was not severe; and (ii) the PVNS symptoms could be alleviated so that the patient would not have substantial arthritic pain remaining after treatment.

Under certain circumstances, including severe joint destruction, moderate bilateral hip destruction, severe pain or functional limitations, the THA was chosen. Overall, selecting THA was based on the combination of the following items: (i) the level of pain (visual analogue scale [VAS] >3.0); (ii) function of the hip joint (Harris hip score [HHS] < 70); and (iii) radiological changes: severe joint destruction.

PVNS included diffuse PVNS and localized PVNS.^10^ The localized PVNS was considered to be chosen for arthroscopic treatment and the treatment of diffuse PVNS depended on the degree of joint destruction and symptoms mentioned before.

The approaches utilized for procedures included anterolateral (22 cases), posterior (eight patients), and transtrochanteric (seven patients) approaches. No patients received adjuvant radiotherapy following the surgery.

### Surgical Technique

All THA and arthroscopy procedures were performed by the same group of experienced orthopedic surgeons at a single institution. For THA, direct anterior approach was adopted and femoral neck osteotomy was performed according to the preoperative plan. After the femoral head was removed, the acetabulum was polished and the acetabular prosthesis was implanted. On the femoral side, the test model was placed and the joint was moved to test its stability. Then the test model was removed and the corresponding femoral stem prosthesis and femoral head were implanted.

For arthroscopic treatment, we established an arthroscopic observation channel and an operation channel through the anteromedial lateral, anterolateral, and anterolateral distal ends of the affected hip for exploration. In the anteromedial lateral approach, an anterolateral and anterolateral distal end, a planer, radio frequency, etc., are placed to clean up the joints to release the soft tissue around the hip joint. Nucleus forceps were used to remove many abnormal lesions, which were sent for pathological biopsy, and the affected synovium was resected. To ensure complete resection, the acetabular cartilage, ligaments, glenoid labrum and femoral head were investigated to confirm no residual lesions. After the resection, the hip cavity was examined and cleaned again to confirm no residual lesions. Finally, the hip joint was thoroughly rinsed, and the wound suture and pressure bandaging was performed.

### Radiological Assessment

Postoperative radiological evaluations were performed on either MRI or CT, depending on patient preference. A sports medicine surgeon and a musculoskeletal radiologist evaluated the preoperative and postoperative radiological imaging. If a discrepancy was noted between the two readers, two musculoskeletal radiologists and one sports medicine surgeon reached a consensus. The postoperative radiological assessment aimed to verify cyst removal and structural stability.

### Clinical Outcome

A standard protocol was carried out to obtain the clinical and radiographic follow‐up at 3, 6, and 12 months and 1 and 2 years and every 5 years thereafter. All patients undergoing THA or arthroscopy completed a standardized questionnaire at each follow‐up. The questionnaires were used to assess the VAS score and HHS pre‐ and post‐operatively.^2^ In addition to HHS and VAS, we also evaluated muscle strength based on the UK Medical Research Council (MRC) muscle strength assessment and hip flexion.

### Statistical Analysis

All the data were analyzed using the Statistical Package for Social Sciences 15.0 for Windows (SPSS Inc., Chicago, IL, USA). The *t* test was used to compare the preoperative and final follow‐up hip function within the THA group and the arthroscopy group and between the two groups respectively. A chi square test was used to compare the categorical variable, *P* values < 0.05 were considered statistically significant.

## Results

### General Results

For the remaining 17 patients in the THA group and 20 patients in the arthroscopy group, the mean follow‐up was 50.71 ± 24.67 months (range 24–94 months) for THA and 50.30 ± 22.46 months for arthroscopy (range 24–90 months). There were six males and 11 females with a mean age of 38.94 ± 13.46 years (range from 23–66) in the THA group, and 10 males and 10 females with a mean age of 34.15 ± 10.24 years (range from 21–57) in the arthroscopy group. The mean body mass index (BMI) in the THA group was 23.79 ± 3.61 kg/m^2^ (range from 18.75 to 30.07) and 21.70 ± 2.06 for the arthroscopy group (range from 18.67 to 25.95). The details are shown in Table 1. Though the BMI of THA was significantly higher than that of arthroscopy, both BMI of the two groups remained in the normal range^11^ (18.5–24.9 kg/m^2^).

**TABLE 1:** Basic information of the patients

Characteristics	THA	Arthroscopy	*P* value
Total number	17	20	
Age	38.94 ± 13.46	34.15 ± 10.24	*t* = −1.229; *P* = 0.227
Gender			
Male	6 (35.3%)	10 (50%)	*P* = 0.508 (Fisher's test)
Female	11 (64.7%)	10 (50%)	
Affected side			
Left	8 (47%)	8 (40%)	*P* = 0.746 (Fisher's test)
Right	9 (93%)	12(60%)	
Preoperative VAS	3.65 ± 0.79	4.05 ± 0.94	*t* = −1.395; *P* = 0.172
Preoperative HHS score	45.24 ± 10.36	45.30 ± 11.08	*t* = −0.018; *P* = 0.986
BMI	23.79 ± 3.61	21.70 ± 2.06	*t* = 2.111; *P* = 0.045
Follow‐up	50.71 ± 24.67	50.30 ± 22.46	*t* = 0.052; *P* = 0.959

*Note*: Data are presented as the means ± SD or numbers (%); *P* > 0.05: statistically nonsignificant (n.s.).

Abbreviations: HHS, Harris hip score; THA, total hip arthroplasty; VAS, visual analog scale.

**Fig. 1:**
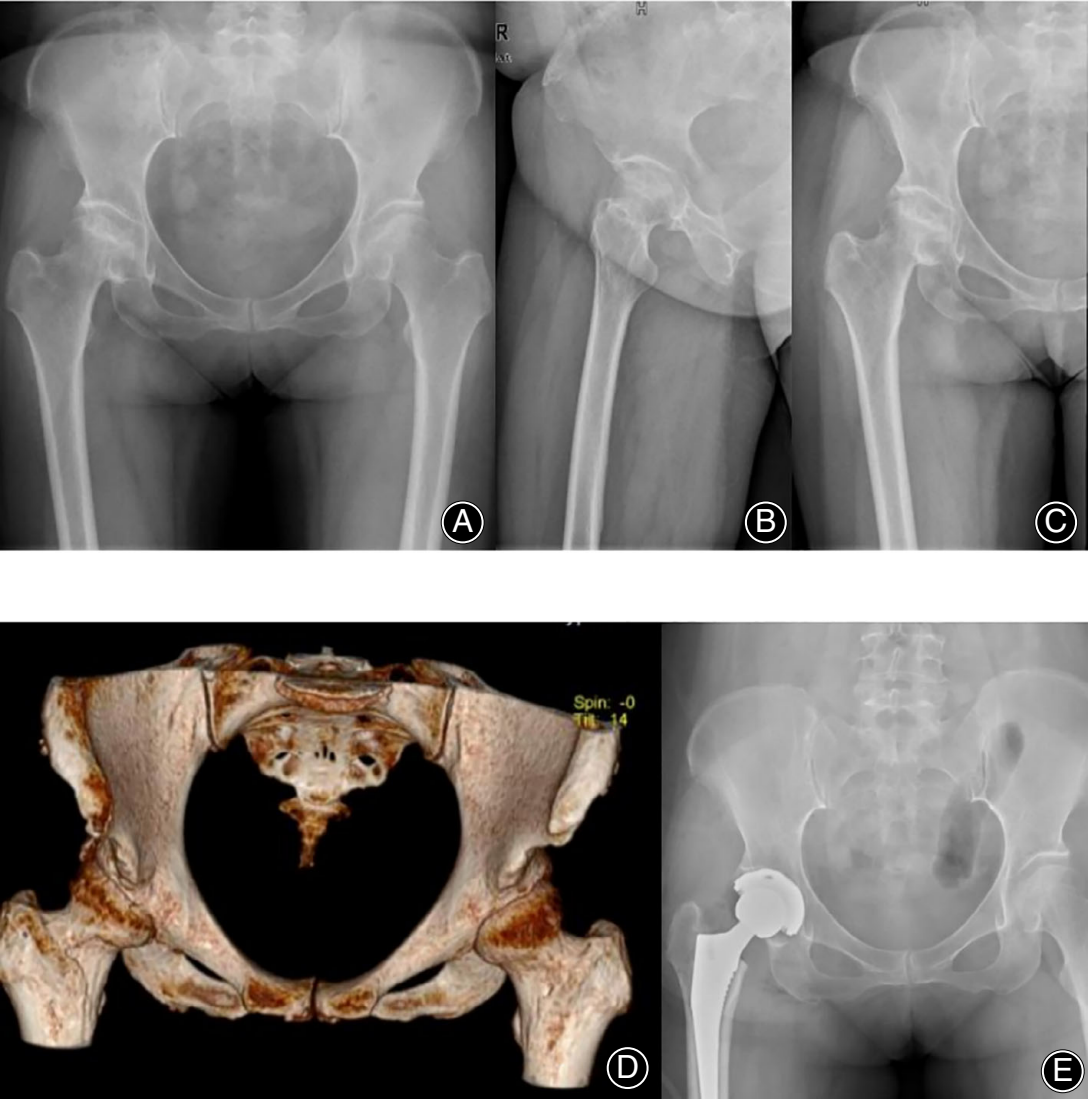
The images of hip PVNS after THA. (A–C) Preoperative pelvic radiograph depicting the right hip to PVNS. (D) Preoperative CT 3D reconstruction of hip PVNS. (E) Postoperative pelvic radiograph

**Fig. 2:**
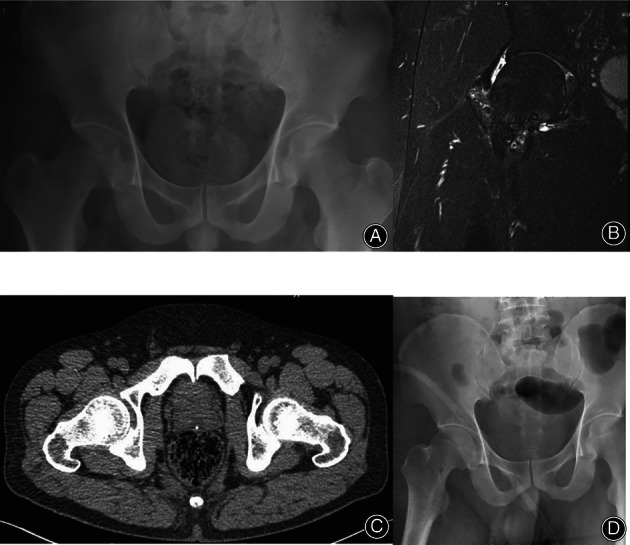
The images of hip PVNS received arthroscopy. (A) Preoperative pelvic radiograph depicting the right hip to PVNS. (B) Preoperative MRI. (C) Preoperative CT 3D reconstruction of hip PVNS. (D) Postoperative pelvic radiograph

**Fig. 3:**
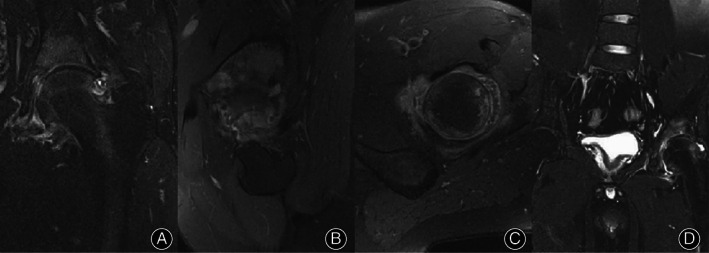
The MRI of hip PVNS received arthroscopy. (A, B, C) Preoperative MRI of the left hip. (D) MRI after operation

After the arthroscopy and radiological assessment (Figs 1, 2, 3), two patients in the arthroscopy group had femoral head necrosis, but symptoms were alleviated after a joint clearing operation. Three patients in the arthroscopy group had hip osteoarthritis, but symptoms were alleviated after a joint clearing operation. For the THA group, six patients had hip osteoarthritis (one patient with developmental dysplasia of the hip; one patient with Otto's disease). All six patients had severe joint destruction.

The information of hospitalization and surgery are summarized in Table 2. Some information was not recorded in earlier surgical records. The results suggested that the hospital stay and amount of bleeding in THA operations were significantly higher than that of arthroscopy.

**TABLE 2:** Information of hospitalization and surgery

Characteristics	THA (number)	Arthroscopy (number)	T value	*P* value
Hospital stay	9.18 d ± 2.96 (n = 17)	6.90 d ± 3.28 (n = 20)	2.219	0.033
Operation duration	123.23 min ± 55.13 (n = 13)	103.06 min ± 38.99 (n = 17)	1.175	0.250
Amount of bleeding in operation	259.29 ml ± 180.32 (n = 14)	29.41 ml ± 28.55 (n = 17)	4.721	0.000

Abbreviation: THA, total hip arthroplasty.

### Clinical Outcome

Before surgery, the mean HHS in the arthroscopy (except for four patients who reached THA) group was 45.30 ± 11.08 (range from 25 to 64) and 45.24 ± 10.36 in the THA group (range from 25 to 63). There was no difference in the HHS (*t* = −0.018; *P* = 0.986). At the final follow‐up, the HHS in the arthroscopy group improved to 71.60 ± 19.78 (t = −5.187, *p* = 0.000, range from 46 to 95) and 78.94 ± 19.11 (*t* = 6.394, *P* = 0.000) in the THA group (range from 46 to 95). There were no significant differences between the two groups (*t* = 1.051, *P* = 0.301) (Table 3). No patients had postoperative hip flexion contractures in the arthroscopy group compared to the 17.6% (3 patients) postoperatively (Fisher's test, *P* = 0.227) in the THA group.

**TABLE 3:** Comparison of clinical outcomes between TKA group and arthroscopy group (Mean ± SD)

	THA	Arthroscopy	T value	*P* value
Improved VAS	3.29 ± 0.99	2.70 ± 1.69	1.329	0.193
Improved HHS	33.71 ± 21.36	26.30 ± 21.38	1.051	0.301

Abbreviations: HHS, Harris hip score; THA, total hip arthroplasty; VAS, visual analogue scale.

The mean VAS improved from 3.65 ± 0.79 to 0.35 ± 0.70 (*t* = 12.890, *P* = 0.000) in the THA group and 4.05 ± 0.94 to 1.35 ± 1.79 (*t* = 5.979, *P* = 0.001) in the arthroscopy group postoperatively. There were no significant differences between the two groups (*t* = 1.329, *P* = 0.193) (Table 3).

All patients reached level 5 muscle strength by the final follow‐up. All the patients' buckling ranges were over 105 degrees. Their internal and external hip rotation was over 15 degrees. Their hip adduction was over 20 degrees, and abduction over 30 degrees.

### Complications and Recurrence

Eleven patients (64.7%) sustained at least one complication in the THA group; three patients (n = 3, 17.6%) sustained aseptic loosening. Complications resulted in procedure revision in five patients. Those requiring revision due to aseptic loosening included the following: three patients, one case of osteolysis secondary to polyethylene wear, and one of painful bipolar hemiarthroplasty, one case of osteolysis secondary to polyethylene wear. Patient survivorship free from any modification in THAs of highly cross‐linked polyethylene (XLPE) was 100% at 5 years.

Recurrence of PVNS was diagnosed in four patients (20%) of the arthroscopy group 5 years after arthroscopy and no recurrence of PVNS was diagnosed in THA group. Synovectomy, debridement of PVNS, and revision arthroplasty were performed for the four patients. No patients underwent amputation, and the limb salvage rate was 100% at the final follow‐up. All four patients sustained at least one complication that led to THA, and aseptic loosening was most common. The mean time to reach THA after arthroscopy was 44.25 ± 6.98 months.

## Discussion

In this study, we assessed the efficacy of arthroscopic removal of PVNS or THA. The present study revealed that arthroscopy and THA yielded satisfying clinical outcomes regarding functional scores and pain relief at the medium‐ to long‐term follow‐up. Both HHS and VAS were significantly higher and all the patients reached normal muscle strength and range of motion (ROM). For the complications and recurrence, 64.7% of patients sustained at least one complication in the THA group and recurrence of PVNS was diagnosed in 20% of patients in the arthroscopy group.

### Mid‐ to Long‐term Outcomes

In this study, the patients undergoing either arthroscopy or THA in the setting of PVNS were relatively young and experienced an elevated rate of complications and revisions; arthroscopy and THA provide a significant improvement in patients' hip function with a low rate of recurrence. In previous studies, both THA and arthroscopy could lead to a reasonable result including functional scores, VAS, muscle strength and ROM.^12^, ^13^, ^14^, ^15^, ^16^, ^17^, ^18^, ^19^ Some studies had shown Harris hip scores that were near or more than 90 after both procedures.^13^, ^15^, ^17^, ^18^, ^19^ In the cases of early diagnosis of PVNS and no severe cartilage involvement, arthroscopy for hip‐saving therapy could lead to reasonable clinical scores.^15^, ^17^ However, there were some differences between the clinical scores and recurrence of the present study and previous studies, which were related to the sample size, patient baseline, and treatment methods.

For the high rate of complications of THA, the influencing factors for revision are complicated, and different patients have different degrees of tolerance to different materials for THA. Patient characteristics, time since THA and surgical approach may also have an influence. Moreover, PVNs may also be a risk factor for revision. The study of Tibbo *et al*. on the outcomes of THA in the setting of PVNs reported that 16 of 25 patients required revision surgery.^12^

### Recurrence

In the present study, only four patients experiencing recurrence 5 years after the arthroscopic procedure and no reproduction in the THA group. Because of the low rate, no specific risk factors could be identified for tumor recurrence. Nevertheless, these data contributed to previous studies suggesting that THA and synovectomy are superior to synovectomy alone in decreasing the local recurrence rate. Tibbo *et al*.^12^ indicated that THA in the setting of PVNS improves patient function with a low rate of local recurrence despite patients' young ages, with 25 patients with PVNS in their study. Della Valle *et al*.^5^ reported a case series with seven patients with PVNS, four patients treated with THA, and synovectomy patients who had no recurrence after 13 years of follow‐up. In contrast, one patient had no radiographic changes 2 years after diagnosis, one developed severe bone loss after 21 years of radiographic follow‐up, and one suffered a recurrence 9 months after synovectomy alone. Another study presented by Botez *et al*.^4^ reported PVNS recurrence rates between 0% and 40% after synovectomy. A systematic review by Levy *et al*.^20^ reported 17.8% recurrence with synovectomy and 3.8% after THA, with no significant difference.

Disease recurrence was limited in the present study. However, the recurrence rates in previous studies are polytropic and have been as high as 45% with synovectomy alone.^21^, ^22^, ^23^ The lower recurrence rates could be related to the small sample size, patient baseline, severe degree of PVNS and treatment methods of this study. When surgical treatment failure or disease recurrence occur, radiotherapy and chemotherapy are viable. Moderate‐dose external radiotherapy (30–35 Gy) has been proven to decrease local recurrence rates in combination with surgical excision at short‐term follow‐ups.^21^ However, radiotherapy may lead to posttreatment fibrosis and even the possibility of late sarcoma transformation. Radioactive colloids and intra‐articular chemotherapies have been used to avoid these complications. Intra‐articular 32P has been proven to provide more than 70% local control with minimal side effects in two small cohorts of short‐term follow‐up.^24^, ^25^, ^26^ Another multicentre retrospective study^25^ evaluated the clinical results of imatinib mesylate in 29 patients with PVNS. Seven patients in this series received surgical resection. The authors reported that 74% of patients had stable disease at the final follow‐up, but six discontinued due to drug toxicity. The authors warn that they must weigh the benefits of lessening disease and drug‐related side effects. In this study, THA and arthroscopy improved the patient's function with a low recurrence rate; surgical treatment may still be the better choice in the setting of PVNS.

### Limitation and Strengths

This study has several limitations. First, this is a retrospective study from a single institution; therefore, it is subject to the limitations inherent to the study design. Second, the study has a small sample size, however, the incidence of PVNS was relatively small. Third, although there was no statistical difference in baseline data except BMI for the two groups and the BMI of both groups remained in the normal range, the comparison of the two groups was to show the treatment effect of different methods, the severity of the PVNS was not equal. Finally, follow‐up MRI scans were limited due to their high expense and a long waiting period. Therefore, an in‐depth comparison of the PVNS was not achieved.

### Conclusion

THA or arthroscopy in the setting of PVNS can improve patients' function and lead to a low rate of local recurrence. In well‐selected patients for each approach based on patients' baseline, level of pain, function of the hip joint and radiological changes, one can expect a reasonable result; although Harris Hip Scores seem more reliable than other methods of diagnosis.

## Ethical Approval

This research study was conducted retrospectively from data obtained for clinical purposes. We obtained approval for this research from the local institutional review board (2019610).

## Informed Consent

This is a retrospective anonymised study collecting data on patients treated for hip PVNS; the identity of the patients cannot be revealed. In this study, there are radiological images of patients from the study cohort. The purpose of these images is to describe and visualize the subject matter. The images are anonymized, so there is no concern for the identity of patients being disclosed. Following this journal's instructions, there is no need in this case to obtain informed consent from specific patients.

## Author Contributions

L carried out all of the follow‐ups of the study and prepared the manuscript. LM and YX contributed to the follow‐ups of the study and analysis of the data. YYNC and XJS participated in designing the study. GC and JL secured funding and were responsible for the study setup. All authors read and approved the final manuscript.
